# Performance evaluation of a novel MBT LipidART module (Bruker Daltonics) for detection of colistin resistance in *Escherichia coli* and *Klebsiella pneumoniae*

**DOI:** 10.1007/s10096-025-05099-4

**Published:** 2025-03-12

**Authors:** Julija Germ, Mateja Pirs

**Affiliations:** Institute of Microbiology and Immunology, Faculty of Medicine, University of Ljubljana, Ljubljana, Slovenia

## Abstract

We evaluated the MBT LipidART module on a MALDI Biotyper® Sirius System (Bruker Daltonics) for the rapid detection of colistin resistance in *Escherichia coli* (EC) and *Klebsiella pneumoniae* (KPN) by analysing lipid A profiles in negative ion mode. Categorical agreement was achieved for 98.3% EC (*N* = 58) and 85.0% KPN (*N* = 40). Challenges included calibration difficulties, limited availability of compatible equipment and issues with mucoid and adherent KPN isolates that yielded invalid results. While MBT LipidART module shows promise as a rapid tool for detection of colistin resistance, its performance was notably better for EC compared to KPN*.*

## Introduction

The treatment of infections caused by carbapenem-resistant (CR) Gram-negative bacilli (GNB) presents significant challenges due to limited therapeutic options. While newer antimicrobial agents are effective against certain groups of CR-GNB, carbapenemase-producing (CP) GNB **–**particularly those harbouring metallo-β-lactamases (MBL) **–** remain a critical concern [1–4]. The prevalence of MBL-producers among CR-GNB varies significantly across regions [5]. In Slovenia, CP Enterobacterales (CPE) prevalence is steadily rising. Particularly concerning is the high prevalence of MBL-producers, including those co-producing MBL and OXA-48, which reached 51% among newly detected patients with CPE from clinical and surveillance samples in 2022 [6].

Colistin remains one of the last-resort antimicrobial agents for treating CPE infections. The reference broth-microdilution (BMD) method requires one day to result [2, 3, 7]. To address this, several rapid methods, such as colorimetric test for the detection of colistin resistance (ColR) have been developed and evaluated [8, 9].

Both plasmid-mediated ColR, identified in 2015 (*mcr*-genes), and chromosomal adaptations contribute to the acquired ColR. These mechanisms involve lipopolysaccharide (LPS) modifications that add cationic moieties to LPS, such as L-Ara4N or pEtN, altering its charge and reducing its affinity for colistin [7, 10].

Since lipid A modification is a primary driver of ColR, analysis of the lipid profile using mass spectrometry (MS) has been proposed as a potential rapid detection method. In the past, lipid A extraction required technically demanding methods and substantial bacterial biomass for sample preparation [11]. Larrouy-Maumus et al. described a simplified protocol for lipid A extraction from intact bacterial cells using a DHB matrix, which was then analysed using MALDI-TOF/TOF 4800 Proteomic Analyser in negative ion mode. Specific peaks for lipid A in *Escherichia coli* (EC) were observed at m/z 1796, representing a significant step towards the routine use of lipid A analysis for ColR detection [12]. Additional peaks were identified in ColR isolates, corresponding to modified LPS moieties, distinguishing between *mcr*-mediated resistance (m/z 1919) and chromosomal adaptations (m/z 1927). More consistent and reliable results were achieved when lipid analysis was performed on then-new MALDI Biotyper® Sirius (Bruker Daltonics, Germany) system, which operates in negative ion mode [13, 14].

The MBT LipidART Module is a novel commercial test for the rapid detection of ColR isolates. The MBT Lipid Xtract Kit™ facilitates the lipid A extraction. The proprietary software operating on the MALDI Biotyper® Sirius system, enables the detection of characteristic mass peaks for colistin-susceptible (ColS-N), chromosomally-encoded ColR (ColR-C), and plasmid-mediated ColR (ColR-P) EC isolates (and later *Klebsiella pneumoniae*, KPN) with automated result analysis. Initially, the module was registered as Research Use Only (RUO) for EC isolates and showed promising results [15]. Recently, the software upgrade enabled also the detection of ColR in KPN isolates [13], although its application requires wider evaluation [16].

We aimed to evaluate MS approach using the MBT LipidART module for rapid detection of ColR EC and KPN isolates compared to the reference BMD.

## Materials and methods

A total of 98 isolates collected between 2018 and 2019 from our laboratory strain collection (Institute of Microbiology and Immunology, Faculty of Medicine, University of Ljubljana) were included. The isolates were identified using MALDI TOF MS (Microflext LT, Bruker Daltonics, Germany). Colistin susceptibility testing was performed in duplicate using Sensititre™ FRCOL BMD plates (Thermo Scientific, USA), results were interpreted according to EUCAST.

The stored isolates were passaged on blood agar plates, and tests were performed on the overnight cultures from the third passage. The study included 58 EC isolates, 32 ColR (15 of which were MCR-1/2-producers from the international collection) and 26 ColS strains, as well as 40 KPN isolates, of which 16 were ColR and 24 ColS [17–20].

ColR isolates (MIC > 2 mg/l) were screened for the presence of *mcr*(1–5) genes using PCR as described [21].

The samples for the MBT LipidART module were prepared according to the manufacturer’s instructions. Briefly, lipid A was extracted from a 10-µL loop of an overnight bacterial culture using the MBT Lipid Xtract Kit™. Using the DHB matrix, the extracted pellet was applied in duplicates onto disposable MBT Biotarget 96 plates. A maximum of eight samples were prepared per run, as recommended by the manufacturer (oral communication). Mass spectra were acquired in linear negative ion mode after manual calibration. The spectra obtained were processed with default parameters using FlexAnalysis v.3.4 software (Bruker Daltonics). Automated interpretation of the mass spectra categorized the isolates as follows: ColS-N, ColR-P, ColR-C.

The results of the MBT LipidART test were compared with the BMD; in case of discordant results, both methods were repeated for verification. If the MBT LipidART yielded invalid result, the test was repeated. Separate evaluations were performed for EC and KPN isolates; categorical agreement (CA), very-major error rate (VME; BMD-resistant, MBT LipidART-susceptible), major-error rate (ME; BMD-susceptible, MBT LipidART-resistant), positive predictive value (PPV) and negative predictive value (NPV) were calculated.

## Results and discussion

A total of 58 EC isolates were included (32 ColR, 26 ColS), 14 were ESBL-producers, none were CR/CPE. Among 40 KPN isolates (16 ColR, 24 ColS), 17 were ESBL-producers, eight isolates were CR and among them six CPE.

The MBT LipidART results for 58 EC were as follows: 26/26 ColS isolates were recognised as ColS-N; 31/32 ColR isolates were detected, 1/32 isolates needed retesting due a discordant result with VME with a colistin MIC of 16mg/l. Among 31/32 remaining ColR, 16/16 chromosomally-encoded ColR isolates were characterised as either ColR-P or ColR-C; 14/14 *mcr-1* ColR were characterised as ColR-P, the single *mcr-2* isolate was recognised as ColR but characterised as ColR-C. CA, PPV and NPV are presented in Table 1. CA for EC isolates was good, discrimination between plasmid and chromosomal resistance was not optimal, especially for chromosomally-encoded ColR. Some studies have also found good performance of MBT LipidART assay for EC [14, 15].

**Table 1:** Performance of the MBT LipidART (Bruker Daltonics) for detection of colistin resistance compared to the reference broth microdilution method for *Escherichia coli* and *Klebsiella pneumoniae*

N (colistin MIC range in mg/l)	*Escherichia coli*	*Klebsiella pneumoniae*	
		*K. pneumoniae*—initial results	*K. pneumoniae* – final results after retesting
	58 (0.5 – 16)	40 (0.25 – 32)	
CA (%)	98.3	70.0	85.0
ME(%)	0	2.5	0
VME(%)	2.7	5.0	2.5
PPV (%)	100	NA	100
NPV (%)	93	NA	92.0
Invalid results (%)	0	22.5	12.5

*N* number, *MIC* minimal inhibitory concentration, *CA* categorical agreement, *ME* major error, *VME* very major error, *PPV* positive predictive value, *NPV* negative predictive value

The MBT LipidART results for KPN were more challenging to interpret as 12/40 isolates needed retesting due to invalid (error, “no peaks found”; 7/12), inconsistent (one of the MBT LipidART tests in duplicate was invalid; 2/12) or discordant results (3/12), of which one was false positive and two were false negative. After retesting, valid results were still not obtained for 5/12 KPN isolates; four ColR and one ColS according to the BMD. Colonies of three of these isolates were visually mucoid and adherent to the agar surface, which may have contributed to suboptimal sample preparation. Among the remaining 7/12 retested isolates, 1/12 ColR (*mcr*(1–5) negative, mechanism of resistance unknown) isolate remained false susceptible (VME) while we obtained BMD concordant results for the remaining 6/12 isolates. These initial false resistant or false susceptible results would not have been retested in a routine testing workflow. CA, PPV and NPV are presented in Table 1. A recently published study also demonstrated that KPN poses greater challenges for MBT LipidART than EC [16].

One drawback of this method is the manual calibration, which is technically more demanding. Challenges with mucoid and false-negative KPN isolates have been previously reported [16]. One possible explanation for failure to detect ColR using MBT LipidART is the presence of resistance mechanisms unrelated to lipid A modification, such as capsule formation or efflux pumps, which could contribute to ColR [22, 23].

In 2016, the direct detection of lipid A on intact GNB using MALDI-TOF MS was described [12]. While the MALDI lipidomics approach for rapid detection of ColR was very promising, it faced significant technical challenges. These included the mandatory use of negative ion mode, which was not available on MALDI-TOF MS instruments employed in routine microbiology laboratories, as well as a complex lipid extraction protocol. A few years later, Bruker introduced MALDI Biotyper® Sirius instrument for routine use in clinical laboratories. This system, capable of operating in negative ion mode, effectively addressed this limitation. In-house detection of lipid A requires a complex extraction protocol and expertise exceeding that of routine clinical laboratory personnel [12]. Our study group also evaluated this approach, however our results were inconsistent due to suboptimal extraction (data not shown). The introduction of MBT LipidART lipid extraction protocol with greatly improved lipid extraction addressed this limitation.

The novel MBT LipidART shows promising results for the rapid detection of ColR with results obtainable within 30 min of hands-on time, providing a significant advantage for workflow efficiency. However, the assay is currently designated for RUO and not yet suitable for clinical implementation.

Already established colorimeric tests (e.g. Rapid Polymyxin™ NP) require no additional equipment making them more accessible [24]. However, if the instrument is available, MBT LipidART is a promising alternative, its main advantage being smaller amount of bacterial colonies needed as well as potential for differentiation of plasmid and chromosomal mechanisms without molecular testing. We have focused on phenotypical ColR without molecular characterisation of resistance mechanisms in our study. Further validation studies of the MBT LipidART are essential to determine its clinical utility. While performance for EC is excellent, its performance for KPN isolates was hindered by sample preparation issues and potential presence of alternative resistance mechanisms. Future developments of the LipidART software and sample preparation protocols could improve its reliability and broaden its applicability in clinical settings.

## Data Availability

No datasets were generated or analysed during the current study.
